# Distinct associations of blood pressure phenotypes with subclinical cerebrovascular disease and coronary artery calcification in Japanese men

**DOI:** 10.1038/s41440-026-02559-y

**Published:** 2026-02-13

**Authors:** Nomin Bayaraa, Yuichiro Yano, Aya Kadota, Nazar Mohd Azahar, Tran Ngoc Hoang Phap, Takashi Hisamatsu, Keiko Kondo, Sayuki Torii, Akira Fujiyoshi, Takayoshi Ohkubo, Akihiko Shiino, Kazuhiko Nozaki, Katsuyuki Miura

**Affiliations:** 1 NCD Epidemiology Research Center, Shiga University of Medical Science, Shiga, Japan; 2 Department of Public Health, Shiga University of Medical Science, Shiga, Japan; 3 Division for University Hospital Development, Mongolian National University of Medical Sciences, Ulaanbaatar, Mongolia; 4 Department of General Medicine, Juntendo University Faculty of Medicine, Tokyo, Japan; 5 Faculty of Health Sciences, Universiti Teknologi MARA, Cawangan Pulau Pinang, Kampus Bertam, Malaysia; 6 National Institutes of Biomedical Innovation, Health and Nutrition, Osaka, Japan; 7 Department of Public Health, Okayama University Graduate School of Medicine, Dentistry and Pharmaceutical Sciences, Okayama, Japan; 8 Department of Hygiene, School of Medicine, Wakayama Medical University, Wakayama, Japan; 9 Department of Hygiene and Public Health, Teikyo University School of Medicine, Tokyo, Japan; 10 Molecular Neuroscience Research Center, Shiga University of Medical Science, Shiga, Japan; 11 Department of Neurosurgery, Shiga University of Medical Science, Shiga, Japan

**Keywords:** Blood pressure phenotypes, Morning hypertension, Home blood pressure, Subclinical cerebrovascular disease, Coronary artery calcification.

## Abstract

Hypertension, encompassing white-coat hypertension (WCH), masked hypertension (MH), and sustained hypertension (SH), is an established risk factor for cardiovascular diseases (CVDs), including atherosclerosis. However, among the general population, findings on which target organ is affected by the different phenotypes of hypertension remain unclear. In this community-based observational study of Shiga Epidemiological Study of Subclinical Atherosclerosis, 740 Japanese men underwent brain magnetic resonance imaging to assess the presence of lacunar infarction, white-matter hyperintensities, microbleeds, and intracranial artery stenosis (ICAS) between 2012 and 2015. They also underwent office blood pressure (BP) measurements, home BP monitoring for at least five consecutive days, and coronary artery calcification (CAC) assessments between 2010 and 2014. The final analysis included 686 participants without a history of CVDs. Of the 686 participants, the mean age ( ± SD) was 68.0 ( ± 8.3) years, and 39.3% were taking antihypertensive medication. In multivariable-adjusted models, each of WCH, MH, and SH was significantly associated with a higher risk of microbleeds compared to normotension. However, the association of WCH with microbleeds was evident only among those on antihypertensive medication (adjusted odds ratio [OR] 6.75 [95% CI 1.83–24.86]) and absent in those not on such medication (adjusted OR 1.20 [95% CI 0.31–4.73]). SH was associated with lacunar infarction, ICAS, and CAC. Among Japanese men, WCH, MH, SH were associated with subclinical cerebrovascular diseases, whereas only SH was associated with CAC. Moreover, any elevated BP phenotype increased the risk of microbleeds. Our findings suggest that different hypertension phenotypes distinctly affect target organs, particularly the brain and heart.

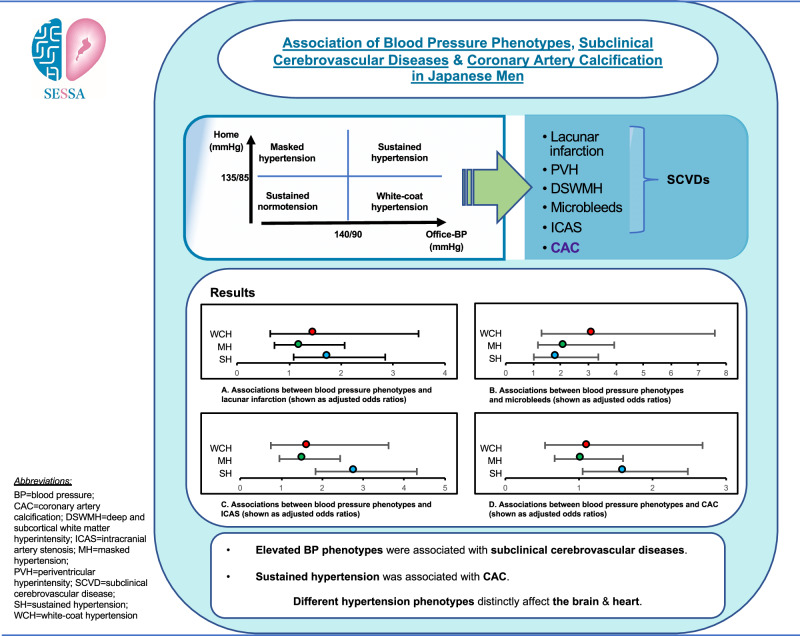

## Introduction

The prevalence of hypertension remains high and still increasing in the East Asian and Pacific regions with the greatest absolute burden of elevated blood pressure (BP) [1, 2]. Hypertension, with its various phenotypes including white-coat hypertension (WCH), masked hypertension (MH), and sustained hypertension (SH), is an established risk factor for cardiovascular diseases (CVDs). WCH is characterized by elevated office BP levels with normal out-of-office BP, while MH is characterized by normal office BP with elevated out-of-office BP findings [3]. Both WCH and MH have been linked to a higher risk of stroke events compared to sustained normal BP, underscoring the challenges in the management of hypertension [4]. Individuals with WCH and MH have higher long-term risks of CVD and total mortality compared to sustained normotension [5, 6], suggesting an independent association between WCH and MH with subclinical CVDs.

Subclinical cerebrovascular diseases (SCVDs) in older adults are highly prevalent and are linked to a greater risk of dementia, stroke, late-life depression, and death, with hypertension being an important vascular risk factor [7]. Moreover, coronary artery calcification (CAC) is a marker of subclinical atherosclerosis and is an independent predictor of myocardial infarction, stroke, and cardiovascular events in the general population [8, 9].

Understanding the associations of different BP phenotypes with SCVDs and CAC is essential for improving cardiovascular risk stratification and developing targeted interventions to prevent the progression of hypertension-related diseases. However, among general population there is still insufficient data on which target organ is affected by the different phenotypes of hypertension. Therefore, we aimed to comprehensively investigate the associations between BP phenotypes and subclinical cerebrovascular diseases (lacunar infarction, deep and subcortical white matter hyperintensity [DSWMH], periventricular hyperintensity [PVH], microbleeds, intracranial artery stenosis [ICAS]) as well as CAC in a Japanese population. Additionally, this study, which included only men, explores whether these associations differ between untreated and treated individuals.

Point of view

- **Clinical relevance**
  Identifying blood pressure phenotypes may facilitate early detection of associated cardiovascular disease risk.
- **Future direction**
  Long-term longitudinal studies are warranted to examine the associations between blood pressure phenotypes and subclinical cerebrovascular diseases and coronary artery calcification in both men and women.
- **Consideration for the Asian population**
  East Asian populations exhibit a higher prevalence of stroke and intracerebral hemorrhage, higher salt intake, and distinct blood pressure profiles compared with Western populations. These characteristics may modify the impact of different blood pressure phenotypes on subclinical cerebrovascular disease and coronary artery calcification.

## Methods

### Study design and population

The Shiga Epidemiological Study of Subclinical Atherosclerosis (SESSA) is an observational study on the factors associated with subclinical atherosclerosis in a community-based sample of randomly selected Japanese residents from Kusatsu City, Shiga, Japan. The characteristics and details of the SESSA study have been previously described [10, 11].

We invited 2379 Japanese men aged 40–79 years to participate in our study based on the Basic Resident Registry of the city. Out of the total number, 1094 men agreed to participate in the baseline examination between 2006 and 2008. Subsequently, 853 participants completed the follow-up examination between 2010 and 2014. All follow-up participants were invited for an MRI examination between 2012 and 2015. A total of 740 participants underwent 1.5 T brain MRI examinations. Out of the 740 participants, we excluded a total of 54 participants due to a history of stroke (*n* = 29), myocardial infarction (*n* = 13), and missing data for exposure and outcome (*n* = 12), leaving 686 individuals for the final analysis (Supplementary Fig. 1). The written informed consent was obtained from all study participants. This study was approved by the Institutional Review Board of Shiga University of Medical Science (R2008-061).

## BP measurements

In the morning, a trained physician applied an appropriately sized cuff on the right arm of each participant. After resting for 5 min while sitting alone in a silent room without crossing the legs or speaking, office BP was measured using an automated sphygmomanometer (BP-8800SF; Omron Healthcare Co. Ltd., Kyoto, Japan) [12]. With reference to the guidelines set by the European Society of Hypertension (ESH), European Society of Cardiology (ESC) [13], and the Japanese Society of Hypertension, office BP was measured twice consecutively within 30-s intervals; the mean of the two BP readings was used for analysis.

Participants were asked to measure their BP at home by themselves using another validated automated device (HEM-705 IT Fuzzy Cuff; Omron Healthcare Co. Ltd.) once in the morning for seven consecutive days, within an hour of awakening, after urination, before having breakfast, before taking any medications and after 2-min of seated relaxation period. Trained staff instructed the study participants on how to use the home BP monitoring device during their clinic visit, where office BP readings were also recorded. Only those who completed a minimum of five consecutive days of home BP monitoring were included in the study. The participant’s mean home BP was calculated by averaging all individual readings.

As a validation of our office BP measurements, we confirmed that office BP was strongly correlated with home BP (*r* = 0.64, *P* < 0.001).

### Cross-classification on the basis of office and home BP

SH was characterized by BP readings of ≥140 mm Hg systolic and/or ≥90 mm Hg diastolic when measured in the office, and ≥135 mm Hg systolic and/or ≥85 mm Hg diastolic when measured at home. Participants receiving antihypertensive medication who exhibit these BP levels were categorized as having sustained uncontrolled BP. WCH was characterized by elevated BP readings in the office setting that are not replicated at home, indicating normal BP levels outside the clinical environment. In cases where medication is used, and the high office BP persists, the condition was referred to as white-coat uncontrolled hypertension (WUCH) [3]. Conversely, MH was defined when a participant’s BP is high at home but normal in the office. If antihypertensive medication was being used under these circumstances, the condition was classified as masked uncontrolled hypertension (MUCH) [3].

Sustained normotension (SN) was defined by consistently normal BP readings in both office and home settings. If these normal readings were maintained with the assistance of medication, the term sustained controlled BP was applied. In cases where systolic and diastolic BP readings fell into different categories—normotensive versus hypertensive—the participant was categorized as hypertensive to reflect the higher risk associated with any consistently high BP reading. Hypertension was also defined according to the AHA/ACC 2017 hypertension guidelines as systolic BP ≥ 130 mm Hg and/or diastolic BP ≥ 80 mm Hg in both office and home BP measurements [14].

### Assessment of covariates

Socio-demographic characteristics and lifestyle factors were collected through a self-administered questionnaire which included data on age, smoking and alcohol consumption status (current, past, or never), medical history (e.g., stroke and myocardial infarction), and medication use (e.g., antihypertensive, antihyperglycemic, and antihyperlipidemic). To ensure accuracy, trained staff reviewed and confirmed these responses with each participant after the questionnaire was completed.

For the analysis of blood parameters, samples were collected from participants after a 12-hour fasting period. These samples were used to measure the concentration of glycated hemoglobin (HbA1c) and serum lipids. HbA1c levels were determined using the latex agglutination inhibition assay (Kyowa Medix, Tokyo, Japan), following the protocols of the Japan Diabetes Society (JDS) or the National Glycohemoglobin Standardization Program (NGSP) and JDS values were converted into NGSP value [15]. Fasting serum lipid concentrations were measured by enzymatic methods and were standardized according to the guidelines provided by the United States Centers for Disease Control and Prevention/Cholesterol Reference Method Laboratory Network. Estimated glomerular filtration rate (eGFR) was calculated using the Japanese Society of Nephrology equation for men [16]: eGFR (ml/min/1.73m^2^)=194×Serum creatinine (mg/dl)^(–1.094)^× age (year)^(–0.287)^.

### Measurement of brain outcomes

Brain MRI and magnetic resonance angiography (MRA) were conducted at Shiga University of Medical Science Hospital from 2012 to 2015, using a 1.5-Tesla MRI machine (Signa HDxt 1.5 T version 16; GE Healthcare, Milwaukee, Wisconsin)[10]. The imaging procedures, including three-dimensional T1-weighted spoiled gradient-recalled (SPGR), two-dimensional T2- and T2*-weighted, fluid-attenuated inversion-recovery (FLAIR), and time-of-flight (TOF) MRA, were utilized to identify small vessel disease and ICAS. The T2- and T2*-weighted, along with FLAIR images, were produced with a 4 mm thickness and without gaps between slices. Two neurosurgeons, KN and AS, accredited by the Japan Neurosurgery Society, independently evaluated all the MRA/MRI images without prior knowledge of the participants’ details. Any differences in their evaluations were resolved through a review by both neurosurgeons.

The gradings of SCVDs were classified as follows: A lacunar infarction was identified as a low signal intensity area on T1-weighted images, measuring between 3 and 15 mm, and appearing as a hyperintense lesion on T2-weighted images. To distinguish these lesions from an expanded perivascular space, the distinctive shape of lacunar infarctions observed in SPGR and the accompanying gliosis seen in FLAIR images were considered. Lacunar infarctions were checked and assigned grades in each specific brain region, including the basal ganglia, brainstem, thalamus, white matter, and other areas, using the following scale: 0, 1 to 2, and ≥3.

White matter hyperintensity (WMH) was characterized as areas of increased signal intensity on FLAIR images and was categorized as either PVH or DSWMH. The classification proposed by Shinohara et al. was then applied to assign grades to PVH and DSWMH[17]. The 2014 Japanese Brain Dock Guidelines included this classification which resembles ones by Fazekas [18, 19].

PVH was graded as follows: no lesions or rims only (grade 0), localized lesions of PVH such as caps (grade 1), enlarged PVH covering the periventricular area (grade 2), diffused PVH extending into deep white matter (grade 3), broad PVH extension covering the whole deep and subcortical white matter (grade 4).

DSWMH was graded as follows: no lesion (grade 0), spotty lesion with a clear boundary and a maximum diameter of less than 3 mm, or an enlarged perivascular cavity (grade 1), patchy and scattered lesion of deep and subcortical white matter with a maximum diameter of more than 3 mm (grade 2), a deep white matter lesion with a fused unclear boundary (grade 3), and largely fused white matter lesion (grade 4).

Microbleeds were characterized as hypointense or demonstrating signal void on T2*-weighted images. The number of microbleeds was counted for several anatomical segments including basal ganglia, cerebellum, cerebral cortex, brainstem, thalamus, and white matter.

Assessment of ICAS was performed in 11 intracranial arteries including the basilar artery with added 5 vessels bilaterally; intracranial segments of the internal carotid artery (ICA); the middle cerebral artery (MCA); anterior cerebral artery (ACA); intracranial segments of the vertebral artery, and the posterior cerebral artery (PCA). Based on the grading system defined in the Warfarin–Aspirin Symptomatic Intracranial Disease study [20], the level of narrowing in each artery was categorized as follows: absence of stenosis, 1–49% stenosis, 50–99% stenosis, and complete occlusion (100%).

### Measurement of coronary artery calcification

The methodology for conducting cardiac CT scans of the SESSA has been formerly reported [10, 11]. CAC was assessed using 16-channel multi-detector row CT (MDCT) using an Aquilion scanner (Toshiba, Tokyo, Japan). Images were captured from the level of the root of the aorta through the heart at a slice thickness of 3 mm, with a scanning duration of 320 ms for MDCT. The images were at 70% of the cardiac cycle, using electrocardiogram triggering during a single breath-hold. The presence of CAC was determined by the identification of at least three contiguous pixels (area = 1 mm²) with a density of ≥130 Hounsfield units (HU), using a DICOM workstation and AccuImage software (AccuImage Diagnostics, South San Francisco, California, USA). A staff trained in CT reading who had no prior clinical information of the participants, read all the CT images and calculated the CAC scores. CAC scores were calculated according to the Agatston method [21].

### Statistical analysis

Descriptive statistics were presented as means (SD) for continuous variables with frequencies and percentages provided where relevant. Dunnett’s test for multiple comparisons was used to test the differences in the mean values and frequencies of risk factors across BP phenotype groups.

Multivariable logistic regression analyses were used to determine the association of BP phenotypes with SCVDs and, separately, with CAC. We dichotomized each of the SCVD outcomes and CAC as follows: lacunar infarction is present (number of lesions ≥1) or absent; PVH is present (grade ≥2) or absent; DSWMH is present (grade ≥3) or absent; microbleeds are present (number of lesions ≥1) or absent; ICAS is present when ≥1% stenosis is identified in any of the arteries assessed or absent; and CAC is present (CAC score >0) or absent.

We ran the analyses using two models. Model 1 was adjusted for age (in years) only. Model 2 was adjusted for age and conventional and behavioral risk factors, including BMI, non-HDL cholesterol, HbA1c, eGFR, as well as smoking and drinking status. All results of the logistic regression models are presented as odds ratios (ORs) with 95% confidence intervals (CIs) to quantify the strength and precision of the associations.

We conducted a subgroup analysis to evaluate the associations of BP phenotypes with SCVDs and CAC, stratified by antihypertensive medication use (participants currently on medication vs. those not on medication). In addition, we performed an interaction analysis to formally test whether the associations between BP phenotypes and outcomes differed by antihypertensive medication status. Interaction was assessed by including cross-product terms between BP phenotypes and antihypertensive medication use in the multivariable regression models.

Sensitivity analysis was performed in 2 approaches. First, we analyzed the associations between BP phenotypes with SCVDs and CAC, excluding anticoagulant users. Second, we analyzed the associations between BP phenotypes with SCVDs and CAC defining BP phenotypes using the 2017 American College of Cardiology (ACC)/American Heart Association (AHA) blood pressure guideline threshold[14].

To facilitate interpretation of differences across BP phenotypes, Wald chi-square tests were used to assess heterogeneity for the overall associations of BP phenotypes categories with each outcome. Corresponding p-values are reported in Tables 2 and 3. All statistical analyses were conducted using SAS software, version 9.4 (SAS Institute, Cary, NC). A two-sided *p* value < 0.05 was considered statistically significant.

## Results

The mean age ( ± SD) of the study participants was 68.0 ( ± 8.3) years, with ages ranging from 46 to 83 years (Table 1). Of the 686 participants, 256 (39.3%) were on antihypertensive medication. Among participants, there were 312 (45.5%) with SN and sustained controlled BP, 35 (5.1%) with WCH and WUCH, 162 (23.6%) with MH and MUCH, and 177 (25.8%) with SH and sustained uncontrolled BP.

**Table 1:** Demographic and clinical characteristics of participants, according to blood pressure phenotypes, SESSA (2010–2014), (*n* = 686), Shiga, Japan

Clinical characteristics	Overall (*n* = 686)	SN (SN and sustained controlled BP) (*n* = 312)	WCH (WCH and WUCH) (*n* = 35)	MH (MH and MUCH) (*n* = 162)	SH (SH and sustained uncontrolled BP) (*n* = 177)
Age, years	68.0 ± 8.3	66.8 ± 8.6	71.7 ± 6.4*	68.5 ± 8.7	68.8 ± 7.5*
BMI, kg/m^2^	23.3 ± 2.9	22.8 ± 2.9	22.5 ± 2.9	23.9 ± 2.6*	23.6 ± 2.9*
Home SBP, mmHg	134.6 ± 16.4	121.5 ± 8.9	127.5 ± 4.6*	143.7 ± 10.6*	150.6 ± 12.6*
Home DBP, mmHg	77.9 ± 10.1	72.3 ± 7.2	72.7 ± 7.1	82.5 ± 8.5*	84.6 ± 10.2*
Office SBP, mmHg	131.7 ± 16.9	120.5 ± 10.1	148.8 ± 7.5*	127.5 ± 7.9*	152.0 ± 12.1*
Office DBP, mmHg	77.1 ± 10.5	71.8 ± 7.8	83.7 ± 7.7*	75.7 ± 7.3*	86.2 ± 10.9*
HDL-C, mg/dL	59.7 ± 16.5	60.0 ± 17.5	61.9 ± 16.5	57.4 ± 15.2	60.7 ± 15.5
LDL-C, mg/dL	118.9 ± 31.5	117.6 ± 31.1	118.8 ± 30.7	118.8 ± 29.7	121.4 ± 33.9
HbA1c, %	5.9 ± 0.8	5.9 ± 0.7	5.7 ± 0.6	5.9 ± 0.8	6.0 ± 0.9
Current smoker, n (%)	134 (19.5)	53 (17.0)	3 (8.6)	38 (23.5)	40 (22.6)
Current drinker, n (%)	555 (80.9)	246 (78.9)	29 (82.9)	126 (77.8)	154 (87.0)
Antihypertensive medication, n (%)	256 (39.3)	98 (31.4)	14 (40.0)	67 (41.4)*	77 (43.5)*
Antidiabetic medication, n (%)	102 (14.9)	36 (11.5)	4 (11.4)	30 (18.5)*	32 (18.1)
Medication for dyslipidemia, n (%)	140 (20.4)	67 (21.5)	8 (22.9)	34 (21.0)	31 (17.5)*

All values are presented as means ± SD for continuous variables. Frequency and percentages are presented for categorical variables

Dunnett’s test for multiple comparisons was used to compare the characteristics among the groups

*P < 0.05 vs. SN and sustained controlled BP group

*BP* indicates blood pressure, *BMI* body mass index, *DBP* diastolic blood pressure, *HbA1c* glycated hemoglobin A1c, *HDL-C* high-density lipoprotein cholesterol, *LDL-C* low-density lipoprotein cholesterol, *MH* masked hypertension, *MUCH* masked uncontrolled hypertension, *SBP* systolic blood pressure, *SN* sustained normotension, *SH* sustained hypertension, *WCH* white-coat hypertension, *WUCH* white-coat uncontrolled hypertension

The clinical characteristics and cardiovascular risk factors were presented according to BP phenotype groups in Table 1. Participants with WCH and SH were significantly older, with mean ages of 71.7 and 68.8 years respectively. Individuals with MH and SH had higher BMI, and a larger proportion of these groups were on antihypertensive medication. Raised home and office systolic and diastolic BP were observed across all groups with elevated BP phenotypes. The SH group had the highest BP.

Table 2 shows the association of four BP phenotypes with SCVDs. In the age adjusted model (Model 1), we observed that participants with SH and sustained uncontrolled BP had a statistically significant association with lacunar infarction (OR 1.80 [95% CI 1.12–2.87]) compared to those with SN and sustained controlled BP. Similar trends were observed for ICAS, with the SH and sustained uncontrolled BP group showing a significant association with ICAS (OR 2.86 [95% CI 1.89–4.33]). MH and MUCH group demonstrated a statistically significant association with ICAS when compared to the reference group, (OR 1.62 [95% CI 1.04–2.53]). After further multivariable adjustment (Model 2), the associations between SH and sustained uncontrolled BP with lacunar infarction and ICAS remained significant (OR 1.76 [95% CI 1.09–2.85]; OR 2.81 [95% CI 1.84–4.31], respectively). However, the associations between MH and MUCH with ICAS were attenuated in Model 2.

**Table 2:** Associations between blood pressure phenotypes and subclinical cerebrovascular diseases, SESSA (2010–2014), (*n* = 686), Shiga, Japan

Brain outcomes	SN (SN and sustained controlled BP) (*n* = 312)	WCH (WCH and WUCH) (*n* = 35)	MH (MH and MUCH) (*n *= 162)	SH (SH and sustained uncontrolled BP) (*n* = 177)	P heterogeneity vs. SN
1. Lacunar infarction					
No. of cases, %	47 (15)	9 (26)	33 (20)	46 (26)	
Model 1	1.00 (reference)	1.41 (0.61-3.27)	1.27 (0.76–2.11)	1.80 (1.12–2.87)*	0.111
Model 2	1.00 (reference)	1.49 (0.64–3.49)	1.22 (0.72–2.07)	1.76 (1.09–2.85)*	0.140
2. PVH					
No. of cases, %	66 (21)	9 (26)	36 (22)	54 (31)	
Model 1	1.00 (reference)	0.91 (0.40–2.08)	0.92 (0.57–1.49)	1.47 (0.95–2.27)	0.217
Model 2	1.00 (reference)	0.96 (0.41–2.21)	1.01 (0.62–1.65)	1.51 (0.97–2.35)	0.261
3. DSWMH					
No. of cases, %	63 (20)	5 (14)	32 (20)	44 (25)	
Model 1	1.00 (reference)	0.56 (0.21–1.51)	0.92 (0.57–1.48)	1.23 (0.79–1.92)	0.390
Model 2	1.00 (reference)	0.56 (0.20–1.51)	0.93 (0.57–1.53)	1.19 (0.75–1.86)	0.475
4. Microbleeds					
No. of cases, %	25 (8)	9 (26)	27 (17)	27 (15)	
Model 1	1.00 (reference)	3.31 (1.39–7.93)*	2.15 (1.20-3.86)*	1.93 (1.08–3.46)*	0.013
Model 2	1.00 (reference)	3.14 (1.30–7.59)*	2.14 (1.17–3.94)*	1.86 (1.02–3.36)*	0.021
5. ICAS					
No. of cases, %	61 (20)	11 (31)	48 (30)	75 (42)	
Model 1	1.00 (reference)	1.54 (0.71–3.35)	1.62 (1.04–2.53)*	2.86 (1.89–4.33)*	<0.0001
Model 2	1.00 (reference)	1.65 (0.75-3.62)	1.53 (0.96–2.44)	2.81 (1.84–4.31)*	<0.0001

Data are displayed as adjusted odds ratios (95% confidence interval)

Model 1: Adjusted for age only

Model 2: Adjusted for age, BMI, non-HDL cholesterol, HbA1c, eGFR, smoking status, and drinking status

*P < 0.05

*BP* indicates blood pressure, *DSWMH*, deep and subcortical white matter hyperintensity, *eGFR* estimated Glomerular Filtration Rate, *HbA1c* glycated hemoglobin A1c, *HDL* high-density lipoprotein, *ICAS* intracranial artery stenosis, *MH* masked hypertension, *MUCH* masked uncontrolled hypertension, *PVH* periventricular hyperintensity, *SH* sustained hypertension, *SN* sustained normotension, *WCH* white-coat hypertension, *WUCH* white-coat uncontrolled hypertension

WCH and WUCH group exhibited a significantly higher risk of cerebral microbleeds compared to SN and sustained controlled BP, with the highest ORs observed in the Model 1 (OR 3.31 [95% CI 1.39–7.93]) and Model 2 (OR 3.14 [95% CI 1.30–7.59]) (P heterogeneity vs. SN = 0.021) (Table 2). A significant interaction between BP phenotype and antihypertensive medication use was observed for this association (P for interaction = 0.0021 vs. SN; Supplementary Table 6). Increased risks were also noted among participants with MH and SH, with both groups demonstrating statistically significant associations across all models.

Table 3 presents the association between BP phenotypes and CAC. In the Model 1, only SH and sustained uncontrolled BP were significantly associated with CAC (OR 1.74 [95% CI 1.14–2.65]), with this association remaining significant in the Model 2 (OR 1.61 [95% CI 1.05–2.48]). Similar observation was found among participants with antihypertensive medications, sustained uncontrolled BP was significantly associated with CAC (OR 2.94 [95% CI 1.29–6.70]) (Supplementary Table 1). However, among participants not taking such medications, this association was not significant (OR 1.32 [95% CI 0.78–2.26]) (Supplementary Table 2).

**Table 3:** Associations between blood pressure phenotypes and coronary artery calcification, SESSA (2010–2014), (*n* = 686), Shiga, Japan

CAC	SN (SN and sustained controlled BP) (*n* = 312)	WCH (WCH and WUCH) (*n* = 35)	MH (MH and MUCH) (*n* = 162)	SH (SH and sustained uncontrolled BP) (*n* = 177)	P heterogeneity vs. SN
No. of cases, %	**191 (61)**	25 (71)	109 (67)	133 (75)	
Model 1	1.00 (reference)	1.15 (0.52–2.54)	1.19 (0.79–1.80)	1.74 (1.14–2.65)*	0.086
Model 2	1.00 (reference)	1.20 (0.54–2.68)	1.04 (0.67–1.60)	1.61 (1.05–2.48)*	0.170

Data are displayed as adjusted odds ratios (95% confidence interval)

Model 1: Adjusted for age only

Model 2: Adjusted for age, BMI, non-HDL cholesterol, HbA1c, eGFR, smoking status, and drinking status

*P value < 0.05

*BP* indicates blood pressure, *CAC* coronary artery calcification, *eGFR* estimated Glomerular Filtration Rate, *HbA1c* glycated hemoglobin A1c, *HDL* high-density lipoprotein, *MH* masked hypertension, *MUCH* masked uncontrolled hypertension, *SH* sustained hypertension, *SN* sustained normotension, *WCH* white-coat hypertension, *WUCH* white-coat uncontrolled hypertension

Supplementary Table 3 demonstrates the association between BP phenotypes and SCVDs among participants with antihypertensive medication. WUCH group was associated with microbleeds (OR 6.75 [95% CI 1.83–24.86]), and sustained uncontrolled BP was significantly associated with ICAS (OR 2.69 [95% CI 1.39–5.21]), while sustained uncontrolled BP was not associated with microbleeds, and MUCH was not associated with ICAS. Conversely, among participants without antihypertensive medication, SH was associated with microbleeds (OR 2.46 [95% CI 1.15–5.25]) and ICAS (OR 2.72 [95% CI 1.51–4.90]), whereas WCH was not associated with microbleeds (OR 1.20 [95% CI 0.31–4.73]) and ICAS (OR 1.62 [95% CI 0.52–4.98]) (Supplementary Table 4).

When defining BP phenotypes according to ACC/AHA blood pressure guideline, we observed associations similar to those found in our primary analysis between BP phenotypes and SCVDs and CAC in both 2 models. Notably, there was no evidence of significant association between MH or MUCH and microbleeds (Supplementary Table 7 and 8).

## Discussion

In this community-based study of Japanese men without apparent CVD, significant associations were observed between elevated BP phenotypes and SCVDs and CAC. WCH, MH and SH were associated with SCVDs. Whereas only SH was associated with CAC. These associations were also independent of conventional and behavioral cardiovascular risk factors. To our knowledge, this study is the first to show relationships of BP phenotypes to SCVDs, ICAS and CAC, simultaneously, in a general population.

To the best of our knowledge, this is the first study to investigate the association of BP phenotypes including SN, WCH, MH, and SH, defined by strictly measured office and self-measured home BP, with CAC in a general population. In this study, office and home BP measurements were used to define BP phenotypes. Office BP was measured twice during a single visit. Although this might raise concerns about reliability, we carefully standardized the measurement procedures, and the validity of these office BP measurements was supported by our analysis showing a strong correlation with home BP. This suggests that the office BP measured in this study accurately reflect home BP and we can rely on the office BP measurements, even though they were obtained from one visit. In our baseline examination of the SESSA [11], strictly measured office BP and home BP also showed strong and consistent relationships with CAC, with no significant heterogeneity observed.

Previous studies have shown that the effect size of target organ damage is significantly higher in individuals with WCH and MH than in those with SN, and it is comparable to that observed in individuals with SH [22–24]. However, few studies have defined the WCH phenotype using home BP measurements instead of ambulatory BP monitoring (ABPM)[25, 26], and these studies have yielded inconsistent results regarding the association between WCH and SCVDs [25, 27]. The Ohasama study quantified the odds ratios for the presence of silent cerebrovascular lesions, including WMHs and lacunar infarcts [26]. It was reported that both MH and SH were associated with lacunar infarcts and WMHs, but not for WCH. In contrast, the present results revealed a similar association of SH and lacunar infarctions, with slight differences in the strength of the association. These differences could be attributed to several factors. First, the Ohasama study encompassed a broader age range and included both genders, which may capture more variability in BP phenotypes and their effects on cerebrovascular outcomes. In contrast, our study focused exclusively on men, which could lead to differing associations due to gender-specific and age-related differences in cerebrovascular outcomes and BP effects. Second, the methodologies for classifying WMHs differed between the two studies: the Ohasama study classified WMH>grade 1, while our study used a more detailed grading system— PVH>grade 2; DSWMH>grade 3—potentially influencing the observed discrepancies.

In this study, we found that WCH was significantly associated with microbleeds. This association was apparent among participants using antihypertensive medication but was not observed in those not taking such medication. Although the direction of the associations remained consistent across the sensitivity and interaction analyses, supporting the overall pattern of our findings, the association is inconclusive due to limited statistical power within analyses. The association of WCH with cerebral microbleeds, particularly in patients on antihypertensive medication, can be attributed to multiple mechanisms, including BP variability [28], arterial stiffness, and the broader impacts of hypertension on cerebral small vessel integrity [29]. Studies suggest that the variability in BP, especially systolic BP, independently predicts the progression of cerebral microbleeds in deep and infratentorial regions [30, 31]. Abnormal flow pulsations exposing small vessels in the brain to raised pressure fluctuations may contribute to the pathogenesis of cerebral microbleeds. In individuals with uncontrolled hypertension, greater arterial stiffness is significantly associated with larger volumes of white matter lesions and the presence of deep or infratentorial microbleeds [29, 32]. The pathophysiology of hypertension effects on the brain includes micro-aneurysms, impaired cerebral autoregulation, and vessel rupturing, leading to cerebral microbleeds and white matter lesions [33, 34]. In our study, cardiovascular risk factors such as age, BMI, and smoking were significantly higher among participants receiving antihypertensive medication compared to those not taking such medication. Additionally, hypertension, diabetes mellitus, and dyslipidemia were more prevalent among participants those on antihypertensive treatment. This phenomenon may have influenced our results, which showed a significant association between WCH and microbleeds in participants taking antihypertensive medication. Further studies are needed to confirm the related mechanism.

According to a previous study, high BP was significantly associated with poorer outcomes in patients with symptomatic ICAS [35]. Similarly, our analysis found that SH was associated with ICAS (Supplementary Table 3). Additionally, we observed that the association of MH with ICAS was significant among untreated individuals (Supplementary Table 4).

Recent evidence highlights the substantial role that hypertension plays in the development of CAC [36]. Also, it has been shown that the association of office BP with CAC was comparable with that of home BP [11]. We found that only SH was significantly associated with CAC, particularly among participants those with antihypertensive medication. Similar to our findings, Diabetes Prevention Program Outcome Study reported that higher CAC loads were observed in participants taking antihypertensive medications [37]. Transient fluctuations in BP may have a more significant impact on cerebrovascular diseases, SH seems to be more closely associated with CAC. This distinction could be attributed to the unique structural and functional characteristics of the vascular beds in the brain and heart. Cerebral blood flow is maintained by small-vessel, frequency-dependent autoregulation that can be overcome by rapid blood-pressure surges [38], whereas coronary blood flow is regulated by extravascular compression, diastolic-dominant perfusion, metabolic demand, and endothelial mechanisms [39], helping explain their differing susceptibilities to transient BP elevations.

In this study, we conducted sensitivity analyses using the 2017 ACC/AHA BP guidelines to define BP phenotypes, allowing for comparison with global studies. Our population consisted of Japanese men, and applying these guidelines helps generalize our findings internationally. The associations between BP phenotypes and SCVDs and CAC were largely consistent with our primary results. However, the significant association between MH and MUCH with microbleeds found in the primary analysis was not observed with the ACC/AHA definitions. This discrepancy could be attributed to the broader population captured by the ACC/AHA guidelines, which lower the threshold for diagnosing hypertension, potentially diluting associations with specific outcomes like microbleeds [40]. These findings underscore the importance of understanding how different BP thresholds affect cardiovascular risk assessment and the need for tailored treatment strategies.

The relationship between hypertension phenotypes and specific sites of atherosclerosis, such as SCVDs and CAC, highlights the heterogeneity in the impact of hypertension on cardiovascular health. This relationship underscores the need for comprehensive cardiovascular risk assessment in individuals with hypertension, considering the specific phenotype of hypertension and its potential association with subclinical disease progression.

Our study has limitations. Firstly, the cross-sectional design restricts our ability to establish causal and longitudinal relationships. Secondly, while we carefully controlled for major known confounders, residual effects from unmeasured confounders could still partly influence our findings. Thirdly, there was a 2-year interval between BP measurements and brain MRI assessments for all participants, which complicates our understanding of how changes in BP may relate to changes in brain measures over this period. Fourthly, the relatively small number of participants in some BP phenotype groups likely reduced statistical power and may have contributed to wide confidence intervals and potential Type II error. Although the direction and magnitude of the associations were generally consistent across analyses, the limited power means that some findings may appear inconclusive and should be interpreted cautiously. Furthermore, as subgroup analyses involve multiple comparisons, the possibility of Type I error cannot be excluded. We emphasize the need for larger, adequately powered studies to validate and extend our observations. Lastly, our study population was limited to Japanese men, which restricts the generalizability of our results to women or non-Japanese populations. However, the homogeneity of the population minimizes potential confounding from cultural and environmental variations, providing a more controlled context for observing the associations of interest.

### Asian perspectives

East Asian populations exhibit a higher prevalence of stroke and intracerebral hemorrhage, higher salt intake, and distinct blood pressure profiles compared with Western populations [41]. These characteristics may modify the impact of different blood pressure phenotypes on subclinical cerebrovascular disease and coronary artery calcification. Therefore, further studies are needed to confirm and extend our findings across diverse Asian populations and other ethnicities.

## Conclusions

Elevated BP phenotypes (WCH, MH, and SH) were associated with SCVDs, whereas only SH was associated with CAC in this study of Japanese men. Additionally, we found that the risk of microbleeds was significantly higher with any elevated BP phenotypes. Moreover, this effect was particularly significant in those with WUCH who were on hypertension medication. Our findings reveal the importance of identifying BP phenotypes to ensure early detection of associated cardiovascular risks and the initiation of preventive strategies to hinder the advancement of subclinical cardiovascular diseases in men.

## Supplementary information

Supplementary Material
