# Supplementary material for: Distinct associations of blood pressure phenotypes with subclinical cerebrovascular disease and coronary artery calcification in Japanese men
Source: Hypertens Res. 2026 Feb 13;49(4):1128–38. doi: 10.1038/s41440-026-02559-y (PMC13050641; doi:10.1038/s41440-026-02559-y)
Supplement: Supplementary file 1 — Supplementary Material [file 41440_2026_2559_MOESM1_ESM.docx]

**Supplementary Material**

**Distinct associations of blood pressure phenotypes with subclinical cerebrovascular disease and coronary artery calcification in Japanese men**

Nomin Bayaraa^1,2,3^; Yuichiro Yano^1,4^; Aya Kadota^1,2^; Nazar Mohd Azahar^1,5^; Tran Ngoc Hoang Phap^6^, Takashi Hisamatsu^7^; Keiko Kondo^1,2^; Sayuki Torii^1,2^; Akira Fujiyoshi^8^; Takayoshi Ohkubo^9^; Akihiko Shiino^10^; Kazuhiko Nozaki^11^; Katsuyuki Miura^1,2^, for the SESSA Research Group

^1^NCD Epidemiology Research Center, Shiga University of Medical Science, Shiga, Japan.

^2^Department of Public Health, Shiga University of Medical Science, Shiga, Japan.

^3^Division for University Hospital Development, Mongolian National University of Medical Sciences, Ulaanbaatar, Mongolia.

^4^Department of General Medicine, Juntendo University Faculty of Medicine, Tokyo, Japan.

^5^Faculty of Health Sciences, Universiti Teknologi MARA, Cawangan Pulau Pinang, Kampus Bertam, Malaysia.

^6^National Institutes of Biomedical Innovation, Health and Nutrition, Osaka, Japan.

^7^Department of Public Health, Okayama University Graduate School of Medicine, Dentistry and Pharmaceutical Sciences, Okayama, Japan.

^8^Department of Hygiene, School of Medicine, Wakayama Medical University, Wakayama, Japan.

^9^Department of Hygiene and Public Health, Teikyo University School of Medicine, Tokyo, Japan.

^10^Molecular Neuroscience Research Center, Shiga University of Medical Science, Shiga, Japan.

^11^Department of Neurosurgery, Shiga University of Medical Science, Shiga, Japan.

**Corresponding author:**

Katsuyuki Miura, MD, PhD

Professor, NCD Epidemiology Research Center,

Shiga University of Medical Science,

Seta-Tsukinowa-cho, Otsu, Shiga, 520-2192,

JAPAN.

Tel： +81-077-548-2191

miura@belle.shiga-med.ac.jp

**CONTENTS**

SESSA Research Group Members……………………………….……………………..……3

**Supplementary Table 1.** Associations between blood pressure phenotypes and coronary artery calcification among participants with antihypertensive medication………………………………………………………………………………………..4

**Supplementary Table 2.** Associations between blood pressure phenotypes and coronary artery calcification among participants without antihypertensive medication……………………………………………………………………………………….5

**Supplementary Table 3.** Associations between blood pressure phenotypes and subclinical cerebrovascular diseases among participants with antihypertensive medication………………………………………………………………………………………..6

**Supplementary Table 4.** Associations between blood pressure phenotypes and subclinical cerebrovascular diseases among participants without antihypertensive medication…………………………………………………………………………………….…7

**Supplementary Table 5.** Associations between blood pressure phenotypes and subclinical cerebrovascular diseases and coronary artery calcification among participants without anticoagulant therapy………………………………….………………. 8

**Supplementary Table 6.** Adjusted p-values for interaction between blood pressure phenotype and antihypertensive medication use for subclinical cerebrovascular diseases and coronary artery calcification………………..………………………………….9

**Supplementary Table 7.** Associations between blood pressure phenotypes and coronary artery calcification using the ACC/AHA Hypertension Guideline threshold………………………………………………………………………….…………….10

**Supplementary Table 8.** Associations between blood pressure phenotypes and subclinical cerebrovascular diseases using the ACC/AHA Hypertension Guideline threshold…………………………………………………………………………………….….11

**Supplementary Table 9.** Categorized Distribution of Microbleeds……………………...12

**Supplementary Figure 1.** Flowchart of the study participants………….……………….13

**SESSA Research Group Members**

Principal investigator: Katsuyuki Miura (Shiga University of Medical Science, Otsu, Shiga). Past-principal investigator: Hirotsugu Ueshima (Shiga University of Medical Science, Otsu, Shiga).

Research members: Yoshihisa Nakagawa, Yasutaka Nakano, Emiko Ogawa, Shinji Kume, Katsutaro Morino, Itsuko Miyazawa, Yoshiyuki Watanabe, Kazuhiko Nozaki, Kazumichi Yoshida, Satoshi Shitara, Ikuo Tooyama, Akihiko Shiino, Masaki Nishimura, Shigeki Banba, Susumu Kageyama, Hisakazu Ogita, Naomi Miyamatsu, Yasuyuki, Nakamura, Yuichiro Yano, Aya Kadota, Keiko Kondo, Sayuki Torii, Takashi Kadowaki, Sayaka Kadowaki, Takahiro Ito, Ayako Kunimura, Hiroyoshi Segawa, Yukiko Okami, Takeshi Shibukawa, Azusa Shima, Mizuki Ohashi, Megumi Kawashima, Yuuichi Sawayama, Yousuke Higo, Maya Oki, Kaori Kitaoka (Shiga University of Medical Science, Otsu, Shiga), Akira Fujiyoshi, Aya Higashiyama (Wakayama Medical University, Wakayama), Tomonori Okamura, Naoko Miyagawa (Keio University, Tokyo), Tatsuya Sawamura (Shinshu University, Matsumoto, Nagano), Yasuharu Tabara (Shizuoka Graduate University of Public Health, Shizuoka), Akira Sekikawa, Emma JM Barinas-Mitchell (University of Pittsburgh, Pittsburgh, PA, USA), Daniel Edmundowicz (Temple University, Philadelphia, PA, USA), Takayoshi Ohkubo (Teikyo University, Tokyo), Atsushi Hozawa (Tohoku University, Sendai, Miyagi), Yoshitaka Murakami (Toho University, Tokyo), Nagako Okuda (Kyoto Prefectural University), Hisatomi Arima (Fukuoka University, Fukuoka), Yoshikuni Kita (Tsuruga Nursing University, Tsuruga, Fukui), Takashi Hisamatsu (Okayama University, Okayama), Masahiko Yanagita, (Doshisha University, Kyotanabe, Kyoto), Seiko Ohno (National Cerebral and Cardiovascular Center, Suita, Osaka), Naoyuki Takashima (Kyoto Prefectural University of Medicine), Takashi Yamamoto (Kohka Public Hospital, Shiga), Koichiro Azuma (Nerima General Hospital, Tokyo), Yoshino Saito (Aino University, Ibaraki, Osaka).

**Supplementary Table 1. Associations between Blood Pressure Phenotypes and Coronary Artery Calcification among Participants with Antihypertensive Medication, SESSA (2010-2014), (n=256)**

| CAC | Sustained controlled BP  (n=98) | WUCH  (n=14) | MUCH  (n=67) | Sustained uncontrolled BP  (n=77) |
| --- | --- | --- | --- | --- |
| No. of cases, % | 71 (72) | 10 (71) | 50 (75) | 66 (86) |
| Model 1 | 1.00 (reference) | 1.00 (0.29-3.52) | 1.20 (0.59-2.47) | 2.42 (1.10-5.32)* |
| Model 2 | 1.00 (reference) | 1.36 (0.37-5.05) | 1.41 (0.65-3.04) | 2.94 (1.29-6.70)* |

Data are displayed as adjusted odds ratios (95% confidence interval).

Model 1: Adjusted for age only

Model 2: Adjusted for age, BMI, non-HDL cholesterol, HbA1c, eGFR, smoking and drinking status.

*P value <0.05.

BP indicates blood pressure; CAC, coronary artery calcification; HbA1c, glycated hemoglobin A1c; HDL, high-density lipoprotein; MH, masked hypertension; MUCH, masked uncontrolled hypertension; SESSA, Shiga Epidemiological Study of Subclinical Atherosclerosis; WUCH, white-coat uncontrolled hypertension.

**Supplementary Table 2. Associations between Blood Pressure Phenotypes and Coronary Artery Calcification among Participants without Antihypertensive Medication, SESSA (2010-2014), (n=430)**

| CAC | SN  (n=214) | WCH  (n=21) | MH  (n=95) | SH  (n=100) |
| --- | --- | --- | --- | --- |
| No. of cases, % | 120 (56) | 15 (71) | 59 (62) | 67 (67) |
| Model 1 | 1.00 (reference) | 1.15 (0.41-3.20) | 1.09 (0.65-1.82) | 1.35 (0.81-2.26) |
| Model 2 | 1.00 (reference) | 1.33 (0.47-3.79) | 1.02 (0.59-1.75) | 1.32 (0.78-2.26) |

Data are displayed as adjusted odds ratios (95% confidence interval).

Model 1: Adjusted for age only

Model 2: Adjusted for age, BMI, non-HDL cholesterol, HbA1c, eGFR, smoking and drinking status.

*P value <0.05.

CAC indicates coronary artery calcification; HbA1c, glycated hemoglobin A1c; non-HDL, high-density lipoprotein; MH, masked hypertension; SESSA, Shiga Epidemiological Study of Subclinical Atherosclerosis; SH, sustained hypertension; SN, sustained normotension; WCH, white-coat hypertension.

**Supplementary Table 3. Associations between Blood Pressure Phenotypes and Subclinical Cerebrovascular Diseases among Participants with Antihypertensive Medication, SESSA (2010-2014), (n=256)**

| **Brain outcomes** | **Sustained controlled BP**  **(n=98)** | **WUCH**  **(n=14)** | **MUCH**  **(n=67)** | **Sustained uncontrolled BP**  **(n=77)** |
| --- | --- | --- | --- | --- |
| **1. Lacunar infarction** |  |  |  |  |
| No. of cases, % | 33 (34) | 5 (36) | 22 (33) | 34 (44) |
| Model 1 | 1.00 (reference) | 1.62 (0.49-5.43) | 0.79 (0.37-1.66) | 1.58 (0.82-3.06) |
| Model 2 | 1.00 (reference) | 1.73 (0.51-5.90) | 0.83 (0.39-1.77) | 1.58 (0.80-3.13) |
| **2. PVH** |  |  |  |  |
| No. of cases, % | 33 (34) | 6 (43) | 22 (33) | 35 (45) |
| Model 1 | 1.00 (reference) | 2.12 (0.65-6.88) | 0.75 (0.35-1.57) | 1.79 (0.94-3.44) |
| Model 2 | 1.00 (reference) | 2.05 (0.62-6.78) | 0.73 (0.34-1.55) | 1.83 (0.94-3.57) |
| **3. DSWMH** |  |  |  |  |
| No. of cases, % | 32 (33) | 3 (21) | 20 (21) | 26 (34) |
| Model 1 | 1.00 (reference) | 0.66 (0.17-2.55) | 0.64 (0.31-1.33) | 0.96 (0.50-1.86) |
| Model 2 | 1.00 (reference) | 0.61 (0.15-2.40) | 0.64 (0.30-1.35) | 0.97 (0.49-1.95) |
| **4. Microbleeds** |  |  |  |  |
| No. of cases, % | 11 (11) | 6 (43) | 14 (21) | 14 (18) |
| Model 1 | 1.00 (reference) | 7.69 (2.17-27.33)* | 1.99 (0.77-5.12) | 1.33 (0.50-3.53) |
| Model 2 | 1.00 (reference) | 6.75 (1.83-24.86)* | 1.94 (0.74-5.10) | 1.33 (0.49-3.59) |
| **5. ICAS** |  |  |  |  |
| No. of cases, % | 37 (38) | 6 (43) | 30 (45) | 43 (56) |
| Model 1 | 1.00 (reference) | 1.87 (0.58-6.00) | 1.18 (0.59-2.33) | 2.50 (1.32-4.71)* |
| Model 2 | 1.00 (reference) | 1.73 (0.52-5.76) | 1.11 (0.55-2.27) | 2.69 (1.39-5.21)* |

Data are displayed as adjusted odds ratios (95% confidence interval).

Model 1: Adjusted for age only

Model 2: Adjusted for age, BMI, non-HDL cholesterol, HbA1c, eGFR, smoking and drinking status.

*P value <0.05.

BP indicates blood pressure; DSWMH, deep and subcortical white matter hyperintensity; HbA1c, glycated hemoglobin A1c; non-HDL, high-density lipoprotein; ICAS, intracranial artery stenosis; MH, masked hypertension; MUCH, masked uncontrolled hypertension; PVH, periventricular hyperintensity; SESSA, Shiga Epidemiological Study of Subclinical Atherosclerosis; WUCH, white-coat uncontrolled hypertension.

**Supplementary Table 4. Association between Blood Pressure Phenotypes and Subclinical Cerebrovascular Diseases among Participants without Antihypertensive Medication, SESSA (2010-2014), (n=430)**

| Brain outcomes | SN  (n=214) | WCH  (n=21) | MH  (n=95) | SH  (n=100) |
| --- | --- | --- | --- | --- |
| 1. Lacunar infarction |  |  |  |  |
| No. of cases, % | 20 (9) | 4 (19) | 18 (19) | 18 (18) |
| Model 1 | 1.00 (reference) | 1.32 (0.39-4.43) | 1.86 (0.91-3.78) | 1.81 (0.89-3.65) |
| Model 2 | 1.00 (reference) | 1.37 (0.40-3.97) | 1.88 (0.89-3.97) | 1.90 (0.91-3.94) |
| 2. PVH |  |  |  |  |
| No. of cases, % | 38 (18) | 3 (14) | 21 (22) | 23 (23) |
| Model 1 | 1.00 (reference) | 0.42 (0.11-1.53) | 1.04 (0.56-1.95) | 1.15 (0.63-2.10) |
| Model 2 | 1.00 (reference) | 0.43 (0.12-1.59) | 1.07 (0.56-2.04) | 1.19 (0.64-2.21) |
| 3. DSWMH |  |  |  |  |
| No. of cases, % | 34 (16) | 2 (10) | 18 (19) | 22 (22) |
| Model 1 | 1.00 (reference) | 0.41 (0.09-1.88) | 1.11 (0.58-2.10) | 1.35 (0.74-2.48) |
| Model 2 | 1.00 (reference) | 0.40 (0.09-1.87) | 1.05 (0.54-2.04) | 1.32 (0.71-2.47) |
| 4. Microbleeds |  |  |  |  |
| No. of cases, % | 16 (7) | 3 (14) | 16 (17) | 18 (18) |
| Model 1 | 1.00 (reference) | 1.36 (0.35-5.24) | 2.16 (1.01-4.59)* | 2.39 (1.15-4.97)* |
| Model 2 | 1.00 (reference) | 1.20 (0.31-4.73) | 2.22 (1.01-4.88)* | 2.46 (1.15-5.25)* |
| 5. ICAS |  |  |  |  |
| No. of cases, % | 31 (14) | 5 (24) | 26 (27) | 36 (36) |
| Model 1 | 1.00 (reference) | 1.46 (0.49-4.37) | 2.06 (1.13-3.74)* | 3.09 (1.76-5.44)* |
| Model 2 | 1.00 (reference) | 1.62 (0.52-4.98) | 1.78 (0.96-3.31) | 2.72 (1.51-4.90)* |

Data are displayed as adjusted odds ratios (95% confidence interval).

Model 1: Adjusted for age only

Model 2: Adjusted for age, BMI, non-HDL cholesterol, HbA1c, eGFR, smoking and drinking status.

*P value <0.05

DSWMH indicates deep and subcortical white matter hyperintensity; HbA1c, glycated hemoglobin A1c; non-HDL, high-density lipoprotein; ICAS, intracranial artery stenosis; MH, masked hypertension; PVH, periventricular hyperintensity; SESSA, Shiga Epidemiological Study of Subclinical Atherosclerosis; SH, sustained hypertension; SN, sustained normotension; WCH, white-coat hypertension.

**Supplementary Table 5. Associations between Blood Pressure Phenotypes and Subclinical Cerebrovascular Diseases among Participants without Anticoagulant Therapy, SESSA (2010-2014), (n=673), Shiga, Japan**

| Brain outcomes | SN  (SN and sustained controlled BP)  (n=308) | WCH  (WCH and WUCH)  (n=34) | MH  (MH and MUCH)  (n=159) | SH  (SH and sustained uncontrolled BP)  (n=172) | P heterogeneity vs. SN |
| --- | --- | --- | --- | --- | --- |
| 1. Lacunar infarction |  |  |  |  |  |
| No. of cases, % | 47 (15) | 9 (26) | 33 (21) | 45 (26) |  |
| Model 1 | 1.00 (reference) | 1.41 (0.61-3.29) | 1.25 (0.75-2.09) | 1.80 (1.12-2.89)* | 0.114 |
| Model 2 | 1.00 (reference) | 1.51 (0.64-3.56) | 1.21 (0.71-2.06) | 1.75 (1.08-2.86)* | 0.150 |
| 2. PVH |  |  |  |  |  |
| No. of cases, % | 66 (21) | 8 (24) | 36 (23) | 51 (30) |  |
| Model 1 | 1.00 (reference) | 0.77 (0.33-1.83) | 0.91 (0.56-1.47) | 1.40 (0.90-2.17) | 0.278 |
| Model 2 | 1.00 (reference) | 0.80 (0.33-1.92) | 0.99 (0.60-1.62) | 1.42 (0.91-2.24) | 0.329 |
| 3. DSWMH |  |  |  |  |  |
| No. of cases, % | 62 (20) | 5 (15) | 32 (20) | 42 (24) |  |
| Model 1 | 1.00 (reference) | 0.58 (0.21-1.57) | 0.94 (0.58-1.52) | 1.21 (0.77-1.90) | 0.475 |
| Model 2 | 1.00 (reference) | 0.57 (0.21-1.56) | 0.94 (0.57-1.54) | 1.15 (0.73-1.83) | 0.561 |
| 4. Microbleeds |  |  |  |  |  |
| No. of cases, % | 25 (8) | 9 (27) | 27 (16) | 25 (15) |  |
| Model 1 | 1.00 (reference) | 3.36 (1.40-8.07)* | 2.05 (1.13-3.70)* | 1.80 (1.00-3.26) | 0.019 |
| Model 2 | 1.00 (reference) | 3.18 (1.31-7.73)* | 2.02 (1.09-3.73)* | 1.70 (0.93-3.11) | 0.032 |
| 5. ICAS |  |  |  |  |  |
| No. of cases, % | 60 (20) | 10 (29) | 47 (30) | 72 (42) |  |
| Model 1 | 1.00 (reference) | 1.39 (0.62-3.10) | 1.61 (1.02-2.52)* | 2.83 (1.86-4.30)* | <.0001 |
| Model 2 | 1.00 (reference) | 1.48 (0.66-3.34) | 1.53 (0.96-2.43) | 2.81 (1.82-4.32)* | <.0001 |

Data are displayed as adjusted odds ratios (95% confidence interval).

Model 1: Adjusted for age only

Model 2: Adjusted for age, BMI, non-HDL cholesterol, HbA1c, eGFR, smoking status, and drinking status.

*P<0.05

BP indicates blood pressure; DSWMH, deep and subcortical white matter hyperintensity; HbA1c, glycated hemoglobin A1c; HDL, high-density lipoprotein; ICAS, intracranial artery stenosis; MH, masked hypertension; MUCH, masked uncontrolled hypertension; PVH, periventricular hyperintensity; SH, sustained hypertension; SN, sustained normotension; WCH; white-coat hypertension; WUCH, white-coat uncontrolled hypertension.

**Supplementary Table 6. Adjusted p-values for Interaction between Blood Pressure Phenotypes and Antihypertensive Medication Use for Subclinical Cerebrovascular Diseases and Coronary Artery Calcification, SESSA (2010-2014), (n=686)**

| Outcomes | P for interaction (global) | P for interaction  (WCH vs. SN) | P for interaction  (MH vs. SN) | P for interaction  (SH vs. SN) |
| --- | --- | --- | --- | --- |
| 1. Lacunar infarction | 0.0030 | 0.0511 | 0.3722 | 0.0009 |
| 2. PVH | 0.0060 | 0.0747 | 0.9558 | 0.0018 |
| 3. DSWMH | 0.6226 | 0.7851 | 0.8899 | 0.1893 |
| 4. Microbleeds | 0.0148 | 0.0021 | 0.2866 | 0.7528 |
| 5. ICAS | 0.0002 | 0.1567 | 0.3365 | <.0001 |
| 6. CAC | 0.0124 | 0.6659 | 0.2330 | 0.0016 |

Data are displayed as interaction terms (p-values).

Model was adjusted for age, BMI, non-HDL cholesterol, HbA1c, eGFR, smoking and drinking status.

CAC indicates coronary artery calcification; DSWMH, deep and subcortical white matter hyperintensity; HbA1c, glycated hemoglobin A1c; HDL, high-density lipoprotein; ICAS, intracranial artery stenosis; MH, masked hypertension; PVH, periventricular hyperintensity; SESSA, Shiga Epidemiological Study of Subclinical Atherosclerosis; SH, sustained hypertension; SN, sustained normotension; WCH, white-coat hypertension.

**Supplementary Table 7. Association between Blood Pressure Phenotypes and Coronary Artery Calcification using the ACC/AHA Hypertension Guideline Threshold, SESSA (2010-2014), (n=686)**

| Vascular outcome | SN and sustained controlled BP  (n=168) | WCH and WUCH  (n=78) | MH and MUCH  (n=113) | SH and sustained uncontrolled BP  (n=327) |
| --- | --- | --- | --- | --- |
| CAC |  |  |  |  |
| No. of cases, % | 98 (58) | 54 (69) | 64 (57) | 242 (74) |
| Model 1 | 1.00 (reference) | 1.53 (0.85-2.75) | 0.83 (0.50-1.37) | 2.01 (1.33-3.03)* |
| Model 2 | 1.00 (reference) | 1.39 (0.76-2.52) | 0.72 (0.43-1.22) | 1.82 (1.19-2.78)* |

Data are displayed as adjusted odds ratios (95% confidence interval).

Model 1: Adjusted for age only

Model 2: Adjusted for age, BMI, non-HDL cholesterol, HbA1c, eGFR, smoking and drinking status.

*P value <0.05.

ACC/AHA indicates American College of Cardiology/American Heart Association; BP, blood pressure; CAC, coronary artery calcification; HbA1c, glycated hemoglobin A1c; non-HDL, high-density lipoprotein; MH, masked hypertension; MUCH, masked uncontrolled hypertension; SESSA, Shiga Epidemiological Study of Subclinical Atherosclerosis; SH, sustained hypertension; SN, sustained normotension; WCH, white-coat hypertension; WUCH, white-coat uncontrolled hypertension.

**Supplementary Table 8. Association between Blood Pressure Phenotypes and Subclinical Cerebrovascular Diseases using the ACC/AHA Hypertension Guideline Threshold, SESSA (2010-2014), (n=686)**

| Brain outcomes | SN and sustained controlled BP  (n=168) | WCH and WUCH  (n=78) | MH and MUCH  (n=113) | SH and sustained uncontrolled BP  (n=327) |
| --- | --- | --- | --- | --- |
| 1. Lacunar infarction |  |  |  |  |
| No. of cases, % | 22 (13) | 13 (17) | 24 (21) | 77 (24) |
| Model 1 | 1.00 (reference) | 1.38 (0.64-2.97) | 1.70 (0.88-3.30) | 2.11 (1.24-3.62)* |
| Model 2 | 1.00 (reference) | 1.44 (0.66-3.12) | 1.63 (0.83-3.20) | 2.10 (1.21-3.65)* |
| 2. PVH |  |  |  |  |
| No. of cases, % | 33 (20) | 14 (18) | 28 (25) | 90 (28) |
| Model 1 | 1.00 (reference) | 0.86 (0.42-1.75) | 1.20 (0.67-2.17) | 1.50 (0.94-2.40) |
| Model 2 | 1.00 (reference) | 0.86 (0.42-1.76) | 1.19 (0.65-2.19) | 1.50 (0.92-2.42) |
| 3. DSWMH |  |  |  |  |
| No. of cases, % | 28 (17) | 13 (17) | 25 (22) | 78 (24) |
| Model 1 | 1.00 (reference) | 0.98 (0.47-2.01) | 1.35 (0.74-2.47) | 1.53 (0.95-2.48) |
| Model 2 | 1.00 (reference) | 0.96 (0.46-2.00) | 1.31 (0.70-2.43) | 1.47 (0.90-2.42) |
| 4. Microbleeds |  |  |  |  |
| No. of cases, % | 12 (7) | 14 (18) | 9 (8) | 53 (16) |
| Model 1 | 1.00 (reference) | 2.80 (1.22-6.43)* | 1.04 (0.42-2.56) | 2.45 (1.26-4.74)* |
| Model 2 | 1.00 (reference) | 2.87 (1.24-6.66)* | 1.04 (0.41-2.61) | 2.42 (1.23-4.76)* |
| 5. ICAS |  |  |  |  |
| No. of cases, % | 31 (18) | 21 (27) | 27 (24) | 116 (35) |
| Model 1 | 1.00 (reference) | 1.59 (0.84-3.02) | 1.29 (0.71-2.33) | 2.39 (1.52-3.78)* |
| Model 2 | 1.00 (reference) | 1.44 (0.75-2.77) | 1.17 (0.64-2.16) | 2.21 (1.38-3.56)* |

Data are displayed as adjusted odds ratios (95% confidence interval).

Model 1: Adjusted for age only

Model 2: Adjusted for age, BMI, non-HDL cholesterol, HbA1c, eGFR, smoking and drinking status.

*P value <0.05.

ACC/AHA indicates American College of Cardiology/American Heart Association; BP, blood pressure; DSWMH, deep and subcortical white matter hyperintensity; HbA1c, glycated hemoglobin A1c; non-HDL, high-density lipoprotein; ICAS, intracranial artery stenosis; MH masked hypertension; MUCH, masked uncontrolled hypertension; PVH, periventricular hyperintensity; SESSA, Shiga Epidemiological Study of Subclinical Atherosclerosis; SN, sustained normotension; SH, sustained hypertension; WCH, white-coat hypertension; WUCH, white-coat uncontrolled hypertension.

**Supplementary Table 9. Categorized Distribution of Microbleeds, SESSA (2010-2014), (n=686), Shiga, Japan**

| **Category** | **n** | **%** |
| --- | --- | --- |
| 0 MBs | 598 | 87.17 |
| 1–4 MBs | 82 | 11.95 |
| ≥5 MBs | 6 | 0.87 |
| **Total** | 686 | 100.00 |

*MB indicates microbleeds*

**Supplementary Figure 1. Flowchart of the Study Participants**


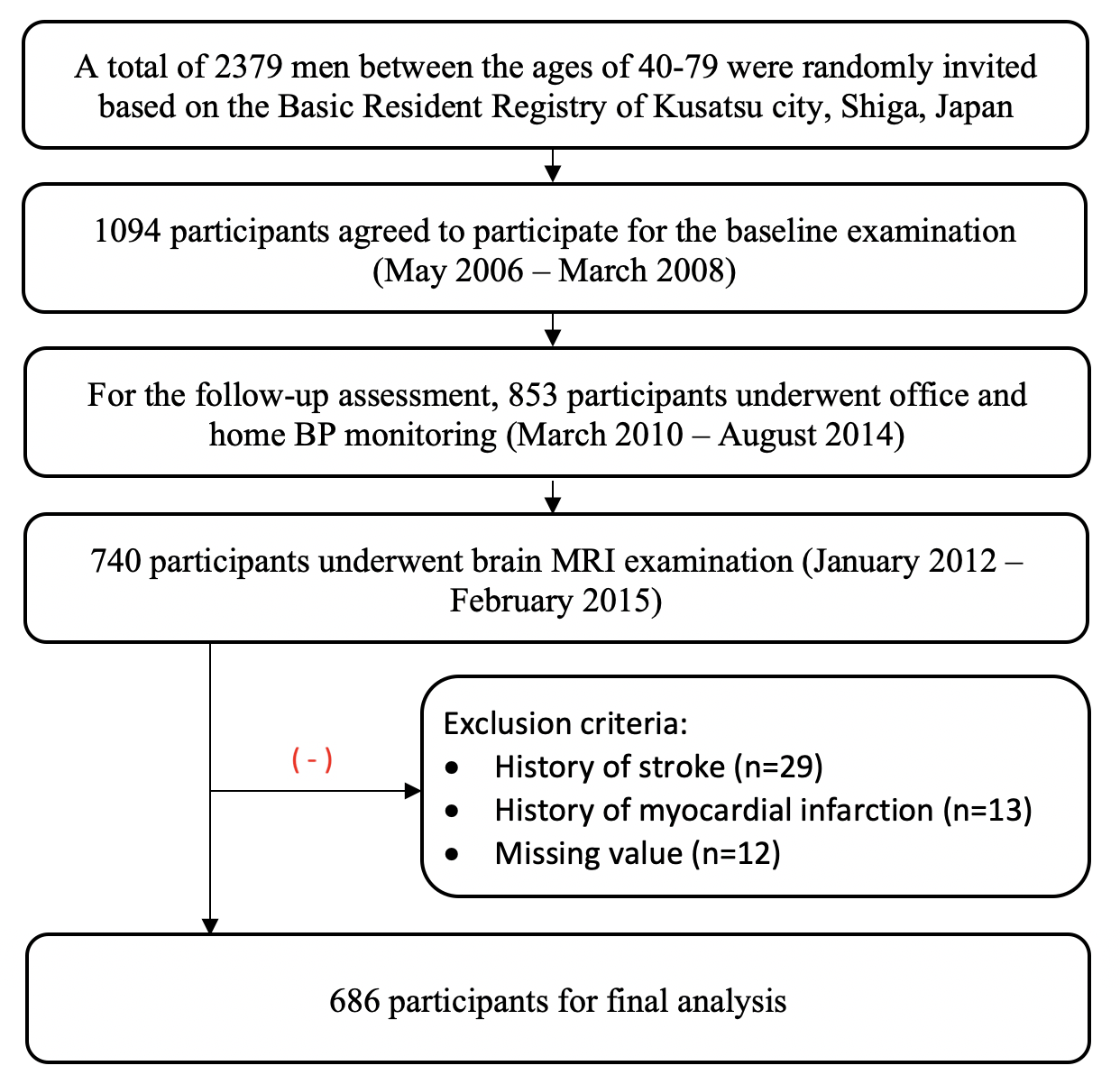


BP indicates blood pressure; MRI, magnetic resonance imaging.
